# Are Electrocardiographic Criteria Reliable for Left Ventricular Hypertrophy Detection in Indian Adults?

**DOI:** 10.7759/cureus.40306

**Published:** 2023-06-12

**Authors:** Bhuvaneswari Kothendaraman, Tiroumourougane Serane V, Kavitha Balasubramanian

**Affiliations:** 1 Department of Medicine, Indira Gandhi Medical College & Research Institute, Puducherry, IND; 2 Department of Pediatrics, A. G. Padmavati's Hospital, Puducherry, IND

**Keywords:** ecg criteria, echocardiography, south indian, left ventricular hypertrophy, 12-lead ecg

## Abstract

Background: Left ventricular hypertrophy (LVH) detection is vital to the risk stratification of adults at risk of adverse cardiovascular events such as coronary heart disease, cerebrovascular disease, and aortic aneurysms. Electrocardiogram (ECG), a non-invasive, cost-effective instrument has been widely used as a screening tool for LVH. The objective of this study was to determine the diagnostic accuracy of seven frequently used ECG criteria in high-risk Indian adults in comparison with echocardiography.

Methods: ECG and transthoracic echocardiography were performed in adults older than 18 years with at least one cardiac risk factor (chronic hypertension, obesity, ischemic heart disease, and type 2 diabetes mellitus). Precision and accuracy were calculated for the various ECG criteria against LVH based on left ventricular mass index (LVMI) and cardiac remodeling by echocardiography.

Results: A total of 220 participants were enrolled. Of these, 96 had LVH by echocardiography. There was marked variability in LVH detection by the different ECG criteria: 28 by Sokolow-Lyon criteria, 26 by Cornell criteria, 24 by Lewis criteria, 46 by Scott criteria, eight by Romhilt-Estes criteria, six by Modified Cornell criteria, and only two by Roberts criteria. Agreement statistics between ECG criteria and LVMI showed that none of them had a good agreement for LVH detection.

Conclusion: None of the ECG criteria were sensitive enough to rule out ventricular hypertrophy. In the context of cardiac remodeling, the ECG criteria had high sensitivity but low specificity and, hence, limited clinical relevance.

## Introduction

Left ventricular hypertrophy (LVH), defined as an increase in left ventricular mass (LVM), is an indicator of left ventricular (LV) pressure or volume overload [1]. LVH is an integral component of cardiovascular risk stratification and is considered to be a surrogate marker of other cardiovascular risk factors integrated over time [2]. It may also contribute directly to cardiovascular disease through pathological changes in cardiac structure and function [3]. The presence of LVH is associated with a rapid increase in the risk of stroke, coronary heart disease, and heart failure. In the Framingham Heart Study, LVH has been associated with a three-fold increase in the incidence of cardiovascular events when compared with a normal heart [4]. Hence, the detection of LVH is integral to the risk stratification of adults at risk of adverse cardiovascular events and other related illnesses [5,6].

LVH can be identified by chest x-ray, electrocardiogram (ECG), echocardiogram, and cardiac MRI. The chest x-ray is neither sensitive nor specific for identifying LVH. Echocardiography has high sensitivity and specificity and is the modality of choice for diagnosing LVH [7]. However, echocardiography is not universally available in resource-poor settings, as it requires vast resources and trained personnel. ECG, a non-invasive, cost-effective instrument has been widely used as a screening tool for LVH. More than 30 criteria-based combinations of parameters of the 12-lead resting ECG have been used for decades to detect LVH [8]. Some of the widely used criteria are Lewis voltage, Gubner-Ungerleider voltage, Romhilt-Estes score, Sokolow-Lyon voltage criteria, Framingham criterion, Perugia criterion, etc. These voltage criteria were developed and validated in Caucasian populations; given the conspicuous racial differences in ECG characteristics, diagnostic accuracy in the Indian population is unproven [9].

The objective of this study was to determine the diagnostic accuracy of seven frequently used ECG criteria for LVH in high-risk Indian adults in comparison with the gold standard, i.e., echocardiography.

## Materials and methods

This prospective analytical study was conducted in a tertiary care hospital, Indira Gandhi Medical College & Research Institute, Puducherry, India. The project proposal for this study (12/158/IEC/PP/2018) was approved by Institute Ethics Committee (Human Studies), Indira Gandhi Medical College & Research Institute. Adults older than 18 years with at least one of the following risk factors, chronic hypertension, obesity, ischemic heart disease, and type 2 diabetes mellitus, were included in the study after informed consent. Patients with acute cardiac illness, rhythm disturbances, coexisting respiratory illness, and in whom a technically adequate LV study could not be performed were excluded from the study. Basic demographic parameters of age, sex, height, weight, abdominal circumference, blood pressure, comorbidities, and medications were entered in a predesigned proforma. Body surface area (BSA), using the Takahira formula, and BMI were calculated [10,11]. Standard 12-lead ECG with standardizations for each of the 12 leads was recorded at 25 mm/s and 10 mm/mV. For LVH detection, the following criteria-based Limb lead voltage (Lewis Score), Precordial lead voltage (Cornell, Modified Cornell, Sokolow-Lyon, and Roberts Criteria), Combination of the limb and precordial voltage (Scott criteria), Combination of voltage and non-voltage (Romhilt-Estes point score) were applied (Appendix 1).

**Figure 1:**
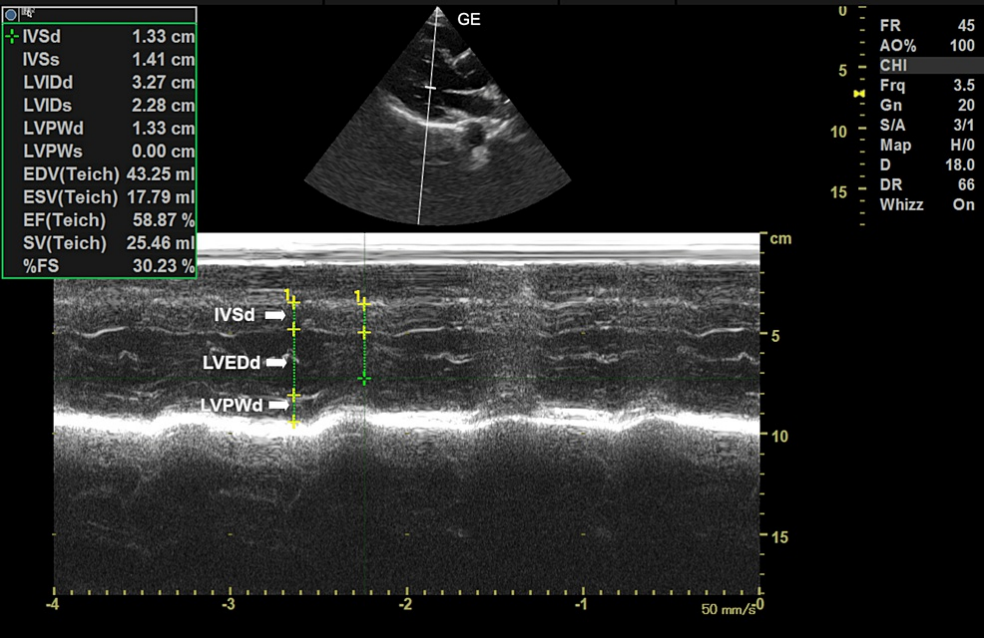
M mode in parasternal long axis view of transthoracic echo showing measurement of various parameters of left ventricular mass LVEDd: LV end diastolic diameter; IVSd: intraventricular septal diameter; LVPWd: LV posterior wall diastolic thickness

Transthoracic echocardiographic examination was performed by a physician who was blinded to the ECG findings and trained in echocardiography, using a Philips HD7XE echocardiogram machine (Koninklijke Philips N.V., Amsterdam, Netherlands). The LV was visualized with the patient lying in a modified left lateral decubitus position, with the probe at the left parasternal window angled to visualize the heart in the long axis view. All the M-mode and two-dimensional (2D) measurements were performed by the leading edge-to-leading edge method, as described by the American Society of Echocardiography (ASE) [12]. Echocardiographic gender-specific grading of ventricular hypertrophy was performed based on the thickness of the interventricular septum, LV posterior wall thickness, and end-diastolic diameter values according to the recommendations of the ASE [13]. LVM was calculated according to the Devereux formula

$$LV mass (g) = 0.8(1.04([LVEDd + IVSd + LVPWd]^{3} - LVEDd^{3})) + 0.6$$

where LVEDd is LV diastolic diameter, IVSd is intraventricular septal diameter, and LVPWd is LV posterior wall diastolic thickness) [14]. (Figure 1)

Relative wall thickness (RWT) was calculated using the formula

$$RWT = 2 &times; LVPWd/LVEDd$$

where LVPWd is posterior wall diastolic thickness and LVEDd is LV diastolic diameter. LVM index (LVMI), another indicator of LVH, was calculated as a ratio of LVM and BSA (Appendix 2).

Data were systematically recorded and analyzed using SPSS Statistics for Windows, Version 17.0 (Released 2008; SPSS Inc., Chicago, United States). Precision and accuracy were calculated for the various ECG criteria against LVH based on LVMI and cardiac remodeling subtypes. Kappa agreement was done to identify the most reliable ECG indicator for diagnosing LVH. Values > 0.75 are considered excellent ones, values < 0.40 as poor concordance, and those between 0.40 and 0.75 as good concordance. Statistical significance was verified in all comparisons by using 95% confidence intervals and a p-value < 0.05.

## Results

**Figure 2:**
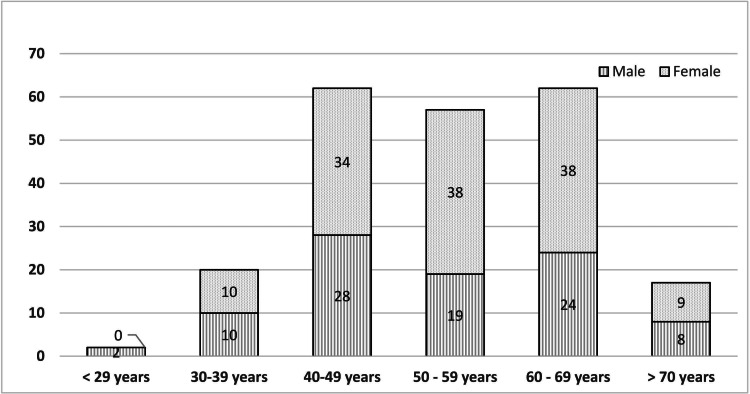
Age and gender-wise distribution of the study population

A total of 220 participants were enrolled in the study; 91 (41.3%) were males and 129 (58.7%) were females. The majority of the subjects were between 40 and 69 years, and the mean age was 53.6 years (Figure 2).

Of the patients, 27% were overweight, and 13.6% were obese based on BMI; 41 males had a waist circumference ≥ 90 cm, while 106 females had a waist circumference ≥ 80 cm. Systolic hypertension was present in 19.1%, while the prevalence of diastolic hypertension was 15.0%.

Table 1 summarises the number of persons with LVH based on various diagnostic criteria. As per echocardiography, 96 had LVH. Of these, 33 (34.4%) were males and the rest 63 (65.6%) were females. Thirty-three (34%) had mild LVH, 26 (27%) had moderate LVH, and 37 (39%) had severe LVH. There was marked variability in presumed LVH detection by the various ECG criteria: 28 by Sokolow-Lyon criteria, 26 by Cornell criteria, 24 by Lewis criteria, 46 patients by Scott criteria, eight by Romhilt criteria, six by Modified Cornell criteria, and two by Roberts criteria.

**Table 1:** Left ventricular hypertrophy as per various diagnostic criteria ECG: Electrocardiograph; LVMI: Left Ventricular Mass Index; LVH: Left Ventricular Hypertrophy; ASE: American Society of Echocardiography

	Male	Female	Total
Echo LVMI			
No LVH	58	66	124
Mild	10	23	33
Moderate	12	14	26
Severe	11	26	37
Echo LVH ASE criteria			
Normal	8	18	26
Concentric remodelling	50	48	98
Concentric hypertrophy	3	4	7
Eccentric hypertrophy	30	59	89
ECG Indices			
Cornell	8	18	26
Modified Cornell	3	3	6
Sokolow-Lyon	18	10	28
Lewis	15	9	24
Scott	28	18	46
Romhilt-Estes	5	3	8
Roberts	1	1	2

**Figure 3:**
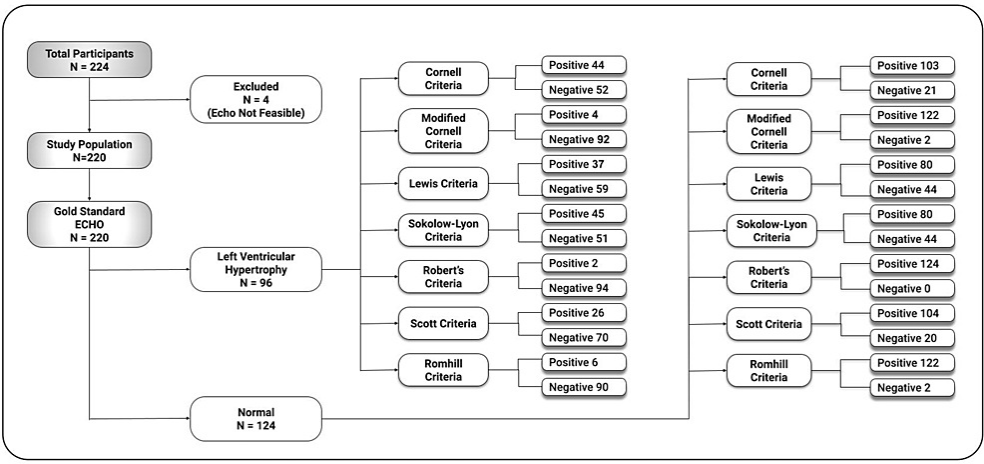
Flow diagram of the diagnostic accuracy of the ECG criteria ECHO: Echocardiography

Figure 3 depicts the flow diagram of the diagnostic accuracy of the various ECG criteria against the gold standard i.e. echocardiogram.

Table 2 shows the precision and accuracy statistics of the various ECG criteria for the detection of LVH by LVMI. Sokolow-Lyon, Robert's, Lewis, and Romhilt criteria had high specificity of > 90%. However, none of the criteria were sensitive enough to rule out ventricular hypertrophy.

**Table 2:** Precision and accuracy statistics (ECG criteria vs LVMI) ECG: Electrocardiograph, LVMI: Left Ventricular Mass Index, PPV: Positive Predictive Value, NPV: Negative Predictive Value

ECG Indices	Sensitivity	Specificity	PPV	NPV
Cornell	19.8%	5.6%	14.0%	8.3%
Modified Cornell	4.2%	1.6%	3.2%	2.1%
Sokolow-Lyon	19.8%	92.7%	67.9%	59.9%
Lewis	13.5%	91.1%	54.2%	57.7%
Scott	27.1%	83.9%	56.5%	59.8%
Romhilt-Estes	6.3%	98.4%	75.0%	57.5%
Roberts	2.1%	100.0%	100.0%	56.9%

Table 3 shows the agreement statistics between ECG criteria and LVMI and none of them had a good agreement for LVH detection.

**Table 3:** Agreement statistics between ECG criteria and echocardiographically defined LVH ECG: Electrocardiograph, LVH: Left Ventricular Hypertrophy, CI: Confidence Interval

	Kappa coefficient	95% CI
Cornell	0.299	0.131
Modified Cornell	0.028	0.148
Sokolow-Lyon	0.114	0.134
Lewis	0.031	0.136
Scott	0.116	0.140
Romhilt-Estes	0.051	0.147
Roberts	0.023	0.149

Table 4 lists the precision and accuracy statistics of the various ECG criteria for the detection of LVH in comparison with cardiac remodeling. All the criteria studied had high sensitivity but low specificity. Thus, none had clinical relevance for LVH detection.

**Table 4:** Precision and accuracy statistics (ECG criteria vs cardiac remodeling) ECG: Electrocardiograph, PPV: Positive Predictive Value, NPV: Negative Predictive Value

	Sensitivity	Specificity	PPV	NPV
Cornell	88.5%	11.9%	11.9%	88.5%
Modified Cornell	96.2%	2.6%	11.7%	83.3%
Sokolow-Lyon	96.2%	13.9%	13.0%	96.4%
Lewis	100.0%	12.4%	13.3%	100.0%
Scott	84.6%	21.6%	12.6%	91.3%
Romhilt-Estes	100.0%	4.1%	12.3%	100.0%
Roberts	100.0%	2.1%	12.0%	100.0%

## Discussion

Among the various risk factors used for cardiovascular risk stratification, LVH is an integral component. ECG is the most frequently employed screening tool for LVH detection. Given the universal availability of ECG, a non-invasive, cost-effective tool, it is appealing to use ECG-based criteria for LVH detection. Since 1949, numerous ECG criteria have emerged based on QRS voltage and duration in limb and precordial leads, in isolation or combination with other features. The oldest criteria formulated by Sokolow and Lyon is presumably the simplest method to predict LVH. Since then, more than 30 ECG criteria have been developed to identify increased LVM from the 12-lead ECG. Some of the routinely used ones include the QRS voltage criteria of Cornell, Cornell voltage-duration product, limb lead criteria of Gubner and Ungerleider, total QRS voltage criteria of Roberts, and the point score of Romhilt and Estes. The reliability and accuracy of the ECG criteria in the detection of LVH have been studied by many and often debated [8,15]. Nevertheless, clinicians have accepted ECG as an essential clinical instrument, despite its perceptible deficiencies.

The accuracy of ECG criteria depends on the prevalence and severity of LVH in the population in which they were developed. Unless tested and proven, the precision of these criteria in our population is questionable due to the inherent difference in the composition and prevalence of LVH. Many studies from high-resource settings have tried to improve the accuracy of ECG criteria for the diagnosis of LVH [16,17]. So far, to the best of our knowledge, very few studies have been conducted to evaluate the reliability and accuracy of various ECG criteria in our population. This research was performed to identify the ECG criterion among the commonly used limb and precordial lead-based voltage criteria, which will be the holy grail of LVH detection in high-risk patients from resource-poor settings.

In the Caucasian population, some studies found that Cornell and Cornell product (CP) criteria performed better than the Sokolow-Lyon index, whereas others delivered conflicting opinions [18]. In our study, none of the ECG criteria demonstrated sensitivity or specificity high enough for reliable detection of anatomically diagnosed LVH. The lack of sensitivity and specificity of widely used ECG criteria make them unacceptable for detecting the presence of echocardiographically confirmed ventricular hypertrophy and, hence, are unsuitable for clinical use in the Indian Adult population.

It is well known that cardiac remodeling is a key aspect of cardiovascular disease progression [19]. Abnormal ECG changes can precede pathological echocardiographic findings, and electrical alterations can add further information to the imaging of cardiac structure [20]. Hence, we studied the reliability and accuracy of the ECG criteria to detect the various types of cardiac remodeling. All the studied criteria demonstrated high sensitivity but low specificity. This shows that though ECG changes do occur in LVH, the commonly used ECG criteria are not clinically useful in detecting the types of cardiac remodeling.

Being a single-center study, the major limitation is the generalizability of the study pending similar research in a larger population. Also, the distribution of the study population was bell-shaped, and more subjects are needed in the extremes of age groups before the conclusions can be generalized.

## Conclusions

Existing ECG criteria should be considered insensitive methods for detecting anatomic LVH in Indian Adults. With reference to cardiac remodeling, the criteria studied had high sensitivity but low specificity and hence, have limited clinical relevance. Therefore, we suggest that further research is required to develop ECG-based criteria for the detection of anatomical LVH in the Indian population.

## Appendices

Appendix 1

**Table 5:** Details of ECG criteria LVH: Left Ventricular Hypertrophy

Criteria				Parameter	
Cornell Criteria				R wave in aVL + S wave in V3 > 28 mm in males or > 20 mm in females	
Modified Cornell Criteria				R wave in aVL >12 mm	
Sokolow-Lyon Criteria				S wave in V1 + R wave in V5 or V6 > 35 mm	
Roberts criteria				QRS voltage in all leads > 175 mm	
Lewis score				Net positivity in lead I + net negativity in lead III ≥ 17 mm	
Scott Criteria	Limb leads			R in I + S in 3 more than 25 mm	
				R in aVL more than 11 mm or >18 mm if left axis is present	
				R in aVF more than 20 mm	
				S in aVR more than 14 mm	
	Chest leads			S in V1 or V2 + R in V5 or V6 more than 35 mm	
				R in V5 or V6 more than 26 mm	
				R + S in any V lead more than 45 mm	
Romhilt-Estes point score	Voltage Criteria (any of)				3
		R or S in limb leads ≥20 mm			
		S in V1 or V2 ≥30 mm			
		R in V5 or V6 ≥30 mm			
	ST-T Abnormalities:				
			ST-T vector opposite to QRS without digitalis		3
			ST-T vector opposite to QRS with digitalis		1
	Negative terminal P mode in V1 1 mm in depth and 0.04 sec in duration (left atrial enlargement)				3
	Left axis deviation (QRS of -30° or more)				2
	QRS duration ≥0.09 sec				1
	Delayed intrinsicoid deflection (interval between beginning of QRS interval and the peak of the R wave) in V5 or V6 (>0.05 sec)				1
	A score of ≥ 5 indicates "definite" LVH; a score of 4 indicates "probable" LVH.				

Appendix 2

**Table 6:** Definition of left ventricular hypertrophy as per left ventricular mass index

Reference Range	Female	Male
Normal	43-95	49-115
Mildly Abnormal	96-108	116-131
Moderately Abnormal	109-121	132-148
Severely Abnormal	≥122	≥149

## References

[1] Nadruz W (2015). Myocardial remodeling in hypertension. J Hum Hypertens.

[2] Gosse P (2005). Left ventricular hypertrophy as a predictor of cardiovascular risk. J Hypertens Suppl.

[3] Bombelli M, Facchetti R, Carugo S (2009). Left ventricular hypertrophy increases cardiovascular risk independently of in-office and out-of-office blood pressure values. J Hypertens.

[4] Agabiti-Rosei E, Muiesan ML (1997). Prognostic significance of left ventricular hypertrophy regression. Adv Exp Med Biol.

[5] (2003). 2003 European Society of Hypertension-European Society of Cardiology guidelines for the management of arterial hypertension. J Hypertens.

[6] Sundström J, Lind L, Arnlöv J, Zethelius B, Andrén B, Lithell HO (2001). Echocardiographic and electrocardiographic diagnoses of left ventricular hypertrophy predict mortality independently of each other in a population of elderly men. Circulation.

[7] Alkema M, Spitzer E, Soliman OI, Loewe C (2016). Multimodality imaging for left ventricular hypertrophy severity grading: a methodological review. J Cardiovasc Ultrasound.

[8] Peguero JG, Lo Presti S, Perez J, Issa O, Brenes JC, Tolentino A (2017). Electrocardiographic criteria for the diagnosis of left ventricular hypertrophy. J Am Coll Cardiol.

[9] Santhanakrishnan R, Wang N, Larson MG (2016). Racial differences in electrocardiographic characteristics and prognostic significance in whites versus asians. J Am Heart Assoc.

[10] Fujimoto S, Watanabe T, Sakamoto A, Yukawa K, Morimoto K (1968). Studies on the physical surface area of Japanese. 18. Calculation formulas in three stages over all ages (Article in Japanese). Nihon Eiseigaku Zasshi.

[11] Keys A, Fidanza F, Karvonen MJ, Kimura N, Taylor HL (2014). Indices of relative weight and obesity. Int J Epidemiol.

[12] Lang RM, Badano LP, Mor-Avi V (2015). Recommendations for cardiac chamber quantification by echocardiography in adults: an update from the American Society of Echocardiography and the European Association of Cardiovascular Imaging. J Am Soc Echocardiogr.

[13] Lang RM, Bierig M, Devereux RB (2005). Recommendations for chamber quantification: a report from the American Society of Echocardiography's Guidelines and Standards Committee and the Chamber Quantification Writing Group, developed in conjunction with the European Association of Echocardiography, a branch of the European Society of Cardiology. J Am Soc Echocardiogr.

[14] Lv T, Yuan Y, Yang J (2021). The association between ECG criteria and Echo criteria for left ventricular hypertrophy in a general Chinese population. Ann Noninvasive Electrocardiol.

[15] Pewsner D, Jüni P, Egger M, Battaglia M, Sundström J, Bachmann LM (2007). Accuracy of electrocardiography in diagnosis of left ventricular hypertrophy in arterial hypertension: systematic review. BMJ.

[16] Chillo P (2021). Sensitivity and specificity of an electrocardiogram to detect echocardiographic left ventricular hypertrophy in a sample of 326 Tanzanian adults: differences in men and women. RRCC.

[17] Marcato JP, Senra Santos F, Gama Palone A, Lenci Marques G (2022). Evaluation of different criteria in the diagnosis of left ventricular hypertrophy by electrocardiogram in comparison with echocardiogram. Cureus.

[18] Snelder SM, van de Poll SW, de Groot-de Laat LE, Kardys I, Zijlstra F, van Dalen BM (2020). Optimized electrocardiographic criteria for the detection of left ventricular hypertrophy in obesity patients. Clin Cardiol.

[19] Angelaki E, Marketou ME, Barmparis GD, Patrianakos A, Vardas PE, Parthenakis F, Tsironis GP (2021). Detection of abnormal left ventricular geometry in patients without cardiovascular disease through machine learning: an ECG-based approach. J Clin Hypertens (Greenwich).

[20] Aro AL, Chugh SS (2016). Clinical diagnosis of electrical versus anatomic left ventricular hypertrophy: prognostic and therapeutic implications. Circ Arrhythm Electrophysiol.

